# Association between Timing of Colonization and Risk of Developing Klebsiella pneumoniae Carbapenemase-Producing K. pneumoniae Infection in Hospitalized Patients

**DOI:** 10.1128/spectrum.01970-21

**Published:** 2022-03-24

**Authors:** Ángela Cano, Belén Gutiérrez-Gutiérrez, Isabel Machuca, Julián Torre-Giménez, Irene Gracia-Ahufinger, Alejandra M. Natera, Elena Pérez-Nadales, Juan Jose Castón, Jesús Rodríguez-Baño, Luis Martínez-Martínez, Julián Torre-Cisneros

**Affiliations:** a Infectious Diseases Unit, Hospital Universitario Reina Sofía-Instituto Maimónides de Investigación Biomédica de Córdoba (IMIBIC)–Universidad de Córdoba, Córdoba, Spain; b Centro de Investigación Biomédica en Red en Enfermedades Infecciosas (CIBERINFEC), Instituto de Salud Carlos III, Seville, Spain; c Infectious Diseases Unit, Hospital Universitario Virgen Macarena –Instituto de Biomedicina de Seville (IBiS)-CSIC, Seville, Spain; d Department of Medicine, Universidad de Seville, Seville, Spain; e Microbiology Unit, Hospital Universitario Reina Sofía-IMIBIC, Córdoba, Spain; f Department of Microbiology, University of Cordoba (UCO), Cordoba, Spain; Houston Methodist Hospital

**Keywords:** carbapenemase-producing *Klebsiella pneumoniae*, timing of colonization, risk of infection

## Abstract

Colonization by KPC-producing Klebsiella pneumoniae (KPC-Kp) is associated with the risk of developing KPC-Kp infection. The impact of the time elapsed since a patient becomes colonized on this risk is not well known. An observational, prospective, longitudinal cohort study of colonized patients undergoing active rectal culture screening to rule out KPC-Kp colonization (July 2012 to November 2017). Patients with a positive culture at inclusion (colonized at start of follow-up) and those with a negative culture at inclusion who became colonized within 90 days (colonized during follow-up) were included in the analysis. CART analysis was used to dichotomize variables according to their association with infection. Kaplan–Meier infection-free survival curves and the log-rank test were used for group comparisons. Logistic regression was used to identify variables associated with KPC-Kp infection. Among 1310 patients included, 166 were colonized at the end of follow-up. Forty-seven out of 118 patients colonized at start of follow-up developed infection (39.8%) versus 31 out of 48 patients colonized during follow-up (64.6%; *P* = 0.006). Variables associated with KPC-Kp infection in the logistic regression analysis were: colonization detection during follow-up (OR, 2.74; 95% CI, 1.07 to 7.04; *P* = 0.03), Giannella risk score (OR, 1.51; 95% CI, 1.32 to 1.73; *P* < 0.001), high-risk ward (OR, 4.77; 95% CI, 1.61 to 14.10; *P* = 0.005) and urological manipulation after admission (OR, 3.69; 95% CI, 1.08 to 12.60; *P* = 0.04). In 25 out of 31 patients (80.6%) colonized during follow-up who developed KPC-Kp infection, infection appeared within 15 days after colonization. The risk of KPC-Kp infection was higher when colonization is recently acquired during hospitalization. In this prospective study, we concluded that the timing of colonization was a factor to assess when considering empirical treatment for suspected KPC-Kp infection and prophylaxis or infection control.

**IMPORTANCE** In this study, it was confirmed that patients who became colonized during hospitalization had a higher risk of developing KPC-Kp infection than hospitalized patients who were already colonized at the start of follow-up. Besides, the risk of infection in the group of patients who became colonized during follow-up was greater in the first weeks immediately after colonization was confirmed. Our findings support the need for designing preventive strategies for patients at the highest risk of infection development, including those admitted in high-risk hospital wards and those undergoing urological procedures.

## INTRODUCTION

Klebsiella pneumoniae carbapenemase-producing K. pneumoniae (KPC-Kp) strains have spread worldwide in recent years (1, 2). KPC-Kp colonization is known to usually precede infection (3, 4). Variables associated with KPC-Kp infection in colonized patients have been extensively investigated (5–8) and scores have been developed to objectively calculate the risk of infection (9–17).

Empirical treatment with coverage of these multidrug-resistant bacteria is initially indicated based on the detection of colonization. Giannella risk score (GRS) (10) is one of the scores used to measure the risk of infection and has been used in combination with the INCREMENT carbapenemase-producing enterobacteriaceae mortality score (INCREMENT-CPE score) score (11) to measure the risk of death in infected patients and to propose a clinical management algorithm (12). These initiatives demonstrate the importance of determining the impact of colonization on the risk of infection in hospitalized patients.

The natural history of intestinal colonization and its clinical impact has been insufficiently studied. It is not known whether the risk of KPC-Kp infection depends on the pathogenic bacterial load or whether it varies with the time elapsed from colonization. This study was designed to investigate whether the risk of KPC-Kp infection is associated with the time elapsed from colonization considering other variables associated with this risk.

## RESULTS

### Patient features.

The overall cohort included 1310 hospitalized patients who were followed during 90 days (except in case of death), of which 166 were colonized at the end of follow-up (78 developed KPC-Kp infection). None uncolonized, unscreened, or patients excluded for any reason developed KPC-Kp infection. Table S1 shows the variables related to the screening protocol.

**FIG 1:**
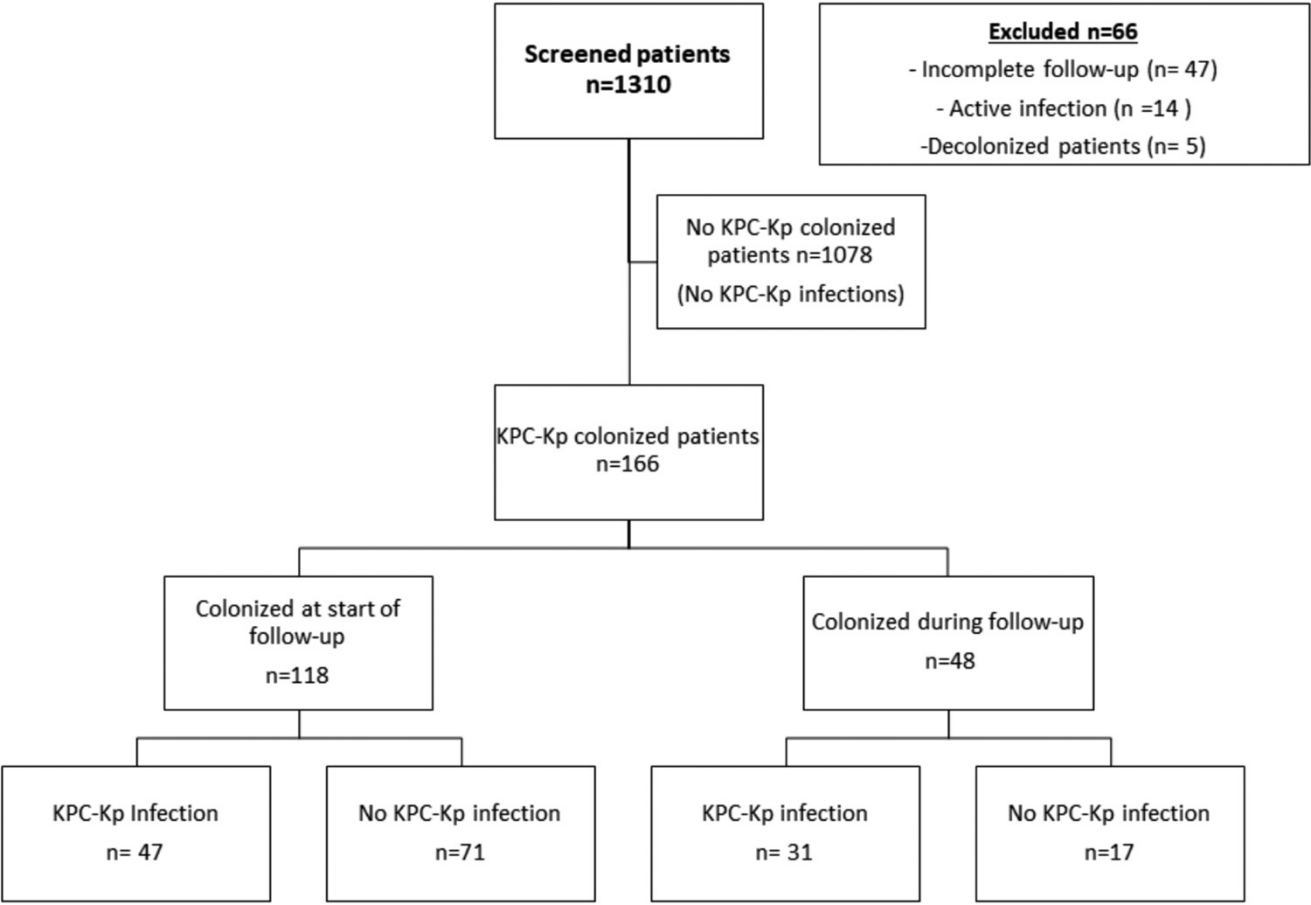
Flow chart.

The classification and regression trees (CART) analysis (Fig. S1) established day 0 of follow-up (colonized at start of follow-up versus colonized during follow-up) as the cutoff point for dichotomizing the variable time to colonization according to the risk of developing KPC-Kp infection. Among 166 colonized patients, 118 (77.1%) were detected by rectal swabs collected at day 0 (colonized at start of follow-up) and 48 (28.9%) by rectal swabs performed within 90 days after the first negative screening (colonized during follow-up) (Fig. 1). Therefore, during the study period, KPC-Kp colonization prevalence was 90 cases/1000 hospitalized patients (118/1310), while the cumulative incidence of KPC-Kp colonization acquired during follow-up was 4.0% (48/1192). The clinical characteristics of these patients, grouped into those colonized at the start of follow-up (day 0) and those colonized during follow-up, are shown in Table 1. In patients colonized during follow-up, the median time (interquartile range) from day 0 to the first positive rectal swab was 1.93 weeks (0.85 to 4.46). Patients colonized at the start of follow-up were older (median, 69 versus 63.50 years; *P* = 0.02) had fewer intensive care unit (ICU) admissions (39.0% versus 68.8%; *P < *0.001) and had a higher Charlson index score (median, 4 versus 2; *P < *0.001). Patients who were colonized during follow-up underwent invasive procedures more frequently, including central venous catheter placement (42.4% versus 83.3%; *P < *0.001), mechanical ventilation (39.5% versus 76.2%; *P < *0.001), or major surgery in both the previous 3 months (42.4% versus 62.5%, *P* = 0.02) and during follow-up (14.4% versus 39.6%, *P* < 0.001). Patients who became colonized during follow-up had a higher GRS than those colonized at the start of follow-up (median, 5 versus 8; *P* = 0.001).

**TABLE 1:** Characteristics of 166 patients with rectal KPC-Kp colonization based on the time when colonization was confirmed during follow-up

Characteristics	Overall^*a*^ (*n* = 166)	Colonized at start of follow-up (*n* = 118)	Colonized during follow-up (*n* = 48)	*P* value^*a*^
Demographics and others				
Age, median (IQR)	68.00 (56.00, 77.00)	69.00 (60.00, 79.00)	63.50 (52.50, 71.00)	0.02^*b*^
Sex, female	59 (35.5)	41 (34.7)	18 (7.5)	0.86
Hospitalization in previous 6 mo	134 (80.7)	94 (79.7)	40 (83.3)	0.67
Intensive care unit admission during follow-up	79 (47.6)	46 (39.0)	33 (68.8)	0.001
High-risk ward	29 (17.5)	26 (22.0)	3 (6.2)	0.01^*c*^
High-risk period (July 2012–June 2014)	74 (44.6)	50 (42.4)	24 (50.0)	0.39
Institutionalization	20 (12.0)	19 (16.1)	1 (2.1)	0.009
Invasive procedures				
Urological manipulation during follow-up	134 (80.7)	91 (77.1)	43 (89.6)	0.08
Central venous catheterization during follow-up	90 (54.2)	50 (42.4)	40 (83.3)	<0.001
Mechanical ventilation during follow-up	64 (52.0)	32 (39.5)	32 (76.2)	<0.001
Major surgery during follow-up	36 (21.7)	17 (14.4)	19 (39.6)	0.001
Major surgery in previous 3 mo	80 (48.2)	50 (42.4)	30 (62.5)	0.02
Upper gastrointestinal endoscopy during follow-up	12 (7.2)	10 (8.5)	2 (4.2)	0.51
Nasogastric intubation during follow-up	35 (21.1)	24 (20.3)	11 (22.9)	0.71
Underlying disease				
Diabetes mellitus	58 (34.9)	47 (39.8)	11 (22.9)	0.05
Heart failure	51 (30.7)	39 (33.1)	12 (25.0)	0.36
Chronic obstructive pulmonary disease	43 (25.9)	36 (30.5)	7 (14.6)	0.05
Kidney disease	34 (20.5)	29 (24.6)	5 (10.4)	0.06
Neoplasia	50 (30.1)	37 (31.4)	13 (27.1)	0.71
Neutropenia	22 (13.3)	16 (13.6)	6 (12.5)	1.00
Charlson index, median (IQR)	3.00 (2.00, 5.00)	4.00 (2.00, 5.00)	2.00 (1.00, 3.00)	<0.001^*a*^
McCabe				0.06
Nonfatal	53 (31.9)	31 (26.3)	22 (45.8)	
Ultimately fatal	84 (50.6)	64 (54.2)	20 (41.7)	
Rapidly fatal	29 (17.5)	23 (19.5)	6 (12.5)	
HIV	3 (1.8)	3 (2.5)	0 (0.0)	0.56^*d*^
Arterial hypertension	94 (56.6)	72 (61.0)	22 (45.8)	0.08
Solid organ transplantation	17 (10.2)	13 (11.0)	4 (8.3)	0.78
Dialysis	16 (9.6)	11 (9.3)	5 (10.4)	0.78
Parenteral drug use	4 (0.4)	4 (3.4)	0 (0.0)	0.32^*c*^
Concomitant treatments				
Steroids	99 (59.6)	65 (55.1)	34 (70.8)	0.08
Antibiotics active against Gram-negative bacilli in previous mo	156 (94.0)	112 (94.9)	44 (91.7)	0.48
Carbapenem treatment in previous mo	65 (39.2)	46 (39.0)	19 (39.6)	1.00
Chemotherapy/radiation in previous 3 mo	12 (7.2)	8 (6.8)	4 (8.3)	0.75
KPC-Kp infection				
Risk of infection in colonized patients according to Gianella risk score, GRS (IQR)	5.00 (5.00, 10.00)	5.00 (5.00, 10.00)	8.00 (5.00, 12.00)	0.01^*b*^
All-site KPC-Kp infection	78 (47.0)	47 (39.8)	31 (64.6)	0.006
Severe KPC-Kp infection (INCREMENT-CPE score > 7)	55 (33.1)	32 (27.1)	23 (47.9)	0.01
KPC-Kp bacteraemia	31 (18.7)	18 (15.3)	13 (27.1)	0.08
Days from colonization confirmation to KPC-Kp infection (IQR) (only patients that developed infection)	6.50 (3.00, 16.5)	10.00 (3.50, 18.50)	4.00 (3.00-11.50)	0.08^*b*^
Mortality				
Crude mortality	69 (41.6)	48 (40.7)	21 (43.8)	0.73
Mortality attributable to KPC-Kp infection	36 (21.7)	23 (19.5)	13 (27.1)	0.30
Mortality not attributable to KPC-Kp infection	33 (19.9)	25 (21.2)	8 (16.7)	0.67
Mortality without having previously developed KPC-Kp infection during follow-up	26 (15.7)	21 (17.8)	5 (10.4)	0.24

a Data presented as number of patients (percentage) except where specified otherwise. *P* values calculated by the Chi-squared test, except where specified otherwise. CPE, carbapenemase-producing *Enterobacterales;* IQR, interquartile range; KPC-Kp, Klebsiella pneumoniae carbapenemase-producing K. pneumoniae; NA, not applicable.

b Mann–Whitney U test.

c Classification by TreeNet.

d Fisher’s exact test.

Forty-seven of the 118 (39.8%) patients colonized at day 0 developed KPC-Kp infection compared to 31 of the 48 (64.6%) patients who were colonized during follow-up (*P* = 0.006). Infections with a higher risk of death (INCREMENT-CPE score >7) occurred in 32 of the 118 (27.1%) patients colonized at the start of follow-up versus 23 of the 48 (47.9%) patients colonized during follow-up (*P* = 0 0.01). No differences in crude or attributable mortality were observed between the two groups. Table S2 shows the distribution of infection types in the two colonization groups.

The KPC-Kp index isolates in our center and some other isolates from this outbreak were previously characterized by multilocus sequence typing (MLST) as corresponding to the same clone (ST512) at the reference laboratory of Virgen Macarena University Hospital of Seville, Spain (18). These isolates were confirmed to be KPC-3 producers and contained *bla*SHV-11 and *bla*TEM-1 genes using PCR with specific primers for class A, B, and D carbapenemases with subsequent sequencing of the obtained amplicons. The isolates showed resistance to ampicillin, cephalosporins, aztreonam, quinolones, amikacin, tobramycin, co-trimoxazole, chloramphenicol, piperacillin-tazobactam, ertapenem, imipenem and meropenem. Resistance rates were variable: fosfomycin (69.6%), gentamicin (53.6%), tigecycline (64.8%) and colistin (66.5%). All tested isolates were sensitive to ceftazidime-avibactam. Sensitivity to ceftazidime-avibactam was determined by disk diffusion using 14 μg discs (Oxoid) from 2015 to February 2017 and by SensititreTM EURGNCOL commercial microdilution panels (Thermo ScientificTM, UK) from March 2017 until the end of the study.

### Adjusted logistic regression analysis of the association between time of colonization and KPC-Kp infection.

The high-risk ward and high-risk period (from July 2012 to June 2014) variables were previously established by Treenet (Fig. S2) and CART (Fig. S3) analyses, respectively, and included in the logistic regression analyses. The multivariate logistic regression analysis (Table 2) confirmed that becoming colonized during follow-up was associated with a higher risk of developing KPC-Kp infection (OR, 2.74; 95% CI, 1.07 to 7.04; *P* = 0.03) in comparison to patients that were colonized at the start of follow-up. Other variables associated with a higher risk of developing KPC-Kp infection were hospitalization in the high-risk ward (OR, 4.77; 95% CI, 1.61-14.10; *P = *0.05), manipulation of the urinary tract (OR, 3.69; 95% CI, 1.08-12.60; *P = *0.04) and GRS (OR per unit, 1.51; 95% CI, 1.32 to 1.73, *P* < 0.001).

**TABLE 2:** Univariate and multivariate logistic regression analysis for KPC-Kp infection among colonized patients^*a*^

Variables	Univariate analysis		Multivariate analysis	
	OR (95% CI)	*P* value	OR (95% CI)	*P* value
Age, per unit	0.98 (0.96–1.00)	0.05		
Female gender	1.57 (0.83–2.98)	0.17		
Prior admission to intensive care unit (previous 6 mo)	1.77 (0.96–3.29)	0.07		
High-risk ward	3.10 (1.63–5.89)	<0.001	4.77 (1.61–14.10)	0.005
High-risk period (July 2012–June 2014)	2.04 (1.10–3.80)	0.02	2.30 (0.98–5.46)	0.06
Institutionalized	0.72 (0.28–1.87)	0.51		
Urological manipulation during follow-up	4.02 (1.63–9.94)	0.002	3.69 (1.08–12.60)	0.04
Central venous catheterization during follow-up	3.62 (1.90–6.92)	<0.001		
Mechanical ventilation during follow-up	3.00 (1.56–5.78)	0.001		
Major surgery during follow-up	1.34 (0.64–2.82)	0.43		
Major surgery in the previous 3 mo	1.54 (0.83–2.83)	0.17		
Nasogastric intubation during follow-up	1.25 (0.59–2.64)	0.55		
Diabetes mellitus	0.63 (0.33–1.21)	0.17		
Heart failure	0.71 (0.37–1.39)	0.32		
Chronic obstructive pulmonary disease	1.11 (0.55–2.21)	0.78		
Kidney disease	1.00 (0.47–2.14)	0.99		
Neoplasia	1.06 (0.54–2.06)	0.86		
Neutropenia	2.19 (0.86–5.54)	0.10		
Charlson index, per unit	0.93 (0.81–1.06)	0.27		
Arterial hypertension	0.73 (0.39–1.36)	0.32		
Solid organ transplantation	1.00 (0.37–2.74)	0.99		
Dialysis	2.73 (0.90–8.23)	0.07		
Chemotherapy/radiation in previous 3 mo	1.64 (0.50–5.38)	0.42		
Gianella risk score (GRS), per unit	1.48 (1.31–1.68)	<0.001	1.51 (1.32–1.73)	<0.001
Colonization during follow-up	2.75 (1.37–5.53)	0.004	2.74 (1.07–7.04)	0.03

a CPE, carbapenemase-producing *Enterobacterales*; KPC-Kp, Klebsiella pneumoniae carbapenemase-producing Klebsiella pneumoniae*;* SHRs: subdistribution hazard ratios.

**FIG 2:**
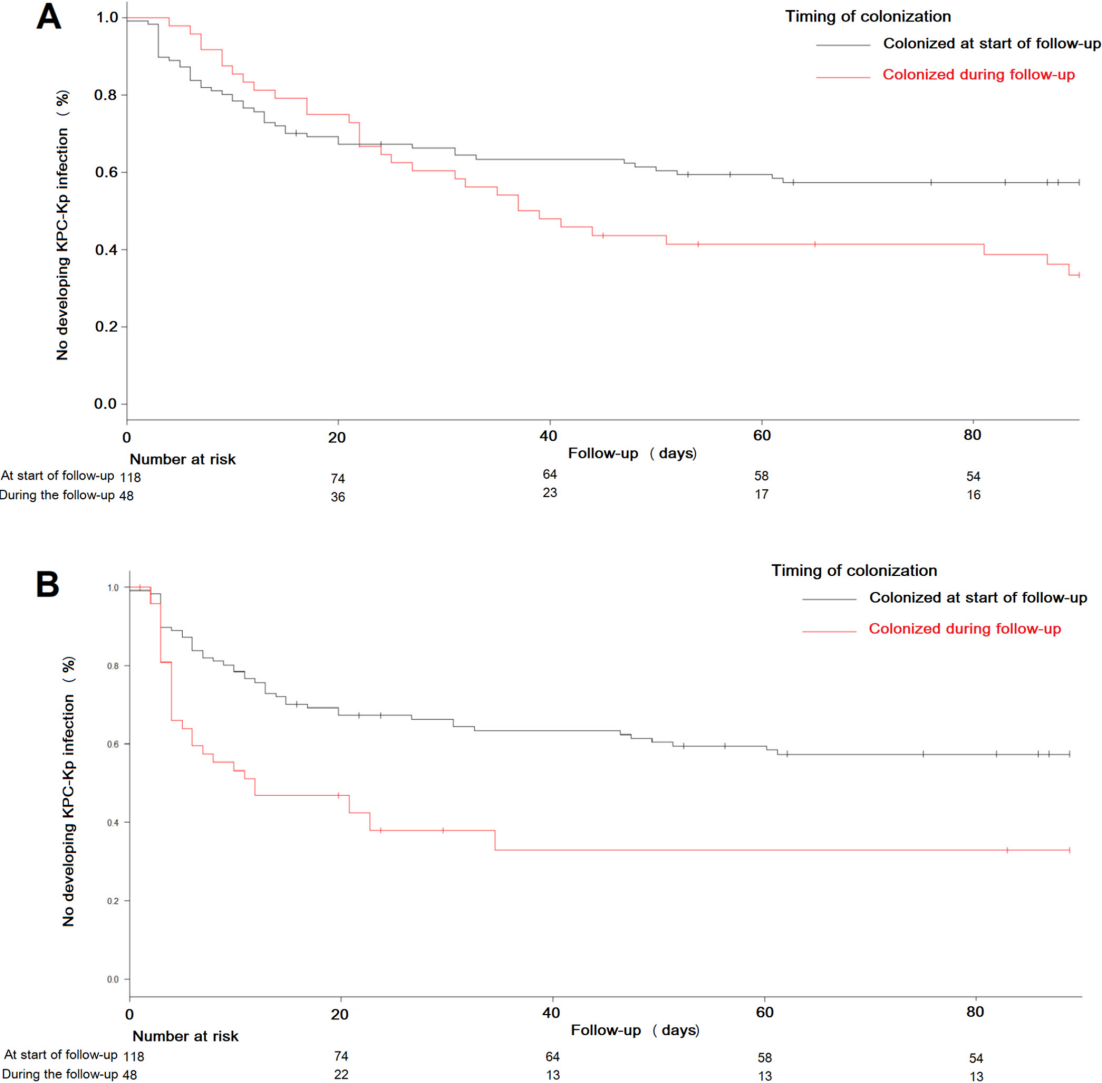
Kaplan–Meier curves of KPC-Kp infection-free survival between patients colonized at start of follow-up and those colonized during follow-up. (A) Considering the start of follow-up from the date of the first rectal swab. (B) Considering the start of follow-up from the date of the first positive KPC-Kp rectal swab. Patients censored before the end of the follow-up period were those who died before developing KPC-Kp infection (see Table 1).

The Kaplan–Meier curves of KPC-Kp infection-free survival, comparing patients colonized at start of follow-up (day 0) and patients colonized during follow-up, confirmed these differences (Fig. 2A, log-rank test *P* = 0.05). Furthermore, the curves showed that the infection-free survival rates decreased in both groups in the first weeks of follow-up until reaching a plateau. Besides, patients colonized during hospitalization developed infections mostly before day 50 of follow-up. The evolution of the number of patients at risk of KPC-Kp infection is shown in Fig. 2A. We observed that 52.1% (25/48) of patients colonized during follow-up developed an infection within the first 40 days. After this time, 26.1% (6/23; *P* = 0.04) of the previously uninfected patients became infected. Fig. 2B shows the Kaplan-Meier curves of KPC-Kp infection-free survival for both groups, considering the moment when colonization by KPC-Kp was confirmed as the start date of follow-up (log-rank test *P* < 0.001). A time window of 30 days with a higher risk of infection can be observed in both groups. We found that 80% (25/31) of the patients colonized during follow-up who finally developed infection became infected within 15 days after colonization was confirmed. These data could not be analyzed in patients colonized at the start of follow-up since it was not possible to know for how long they had been colonized. This suggests the hypothesis that there is a period in the first days after colonization when the risk of developing KPC-Kp infection is higher. The characteristics of these patients with respect to those who did not develop an infection or developed it later are shown in Table 3. Therefore, patients who developed infection before 15 days had a higher GRS (median, 10 versus 5; *P* = 0.001) with higher mortality attributable to infection (44.0% versus 8.7%; *P* = 0.009) than those who developed infection later or did not develop an infection.

**TABLE 3:** Characteristics of patients colonized during follow-up according to whether they developed an infection after 15 days of colonization

Characteristics	Patients in whom the time from colonization to KPC-Kp infection was >15 days or without infection during follow-up (n = 23)	Patients in whom the time from colonization to KPC-Kp infection was ≤15 days (n = 25)	*P* value
Age, median (IQR)	63.00 (25.00, 91.00)	64.00 (19.00, 81.00)	0.765
Sex, female	7 (30.4)	11 (44.0)	0.383
High-risk period (July 2012–June 2014)	11 (47.8)	13 (52.0)	1.000
High-risk service	3 (13.0)	0 (0.0)	0.102
Dialysis	0 (0.0)	5 (20.0)	0.051
Charlson index, median (IQR)	2.00 (0.00, 7.00)	2.00 (0.00, 7.00)	0.469
Urological manipulation during follow-up	20 (87.0)	23 (92.0)	0.660
Neutropenia	3 (13.0)	3 (12.0)	1.000
Giannella risk score, median (IQR)	5.00 (0.00, 20.00)	10.00 (2.00, 22.00)	0.001
All-site KPC-Kp infection	6 (26.1)	25 (100.0)	<0.001
Severe KPC-Kp infection (INCREMENT-CPE score >7)	4 (17.4)	19 (76.0)	<0.001
Crude mortality	9 (39.1)	12 (48.0)	0.22
Mortality not attributable to KPC-Kp infection	7 (30.4)	1 (4.0)	0.020
Mortality attributable to KPC-Kp infection	2 (8.7)	11 (44.0)	0.009

## DISCUSSION

Our study, which was performed in a single center in a situation of the outbreak and subsequent endemicity, shows that the risk of infection was higher in patients who became colonized during hospitalization (colonized during follow-up) than those who were already colonized at the time the first rectal swab screening was performed (colonized at start of follow-up). Furthermore, it shows that the risk of infection in patients colonized during follow-up is higher in the first weeks after colonization is detected until a plateau is reached.

Studies on the kinetics of intestinal colonization are needed to determine whether this time window of increased risk corresponds to the quantitative evolution of the kinetics of intestinal colonization or whether it simply reflects the fact that hospitalization increases the probability that the colonizing bacteria will cause infection. What seems important to us is that we have identified a time window for higher infection risk that allows us to design preventive strategies in certain groups of patients temporally limited to the duration of this time window. For example, we have observed that a high GRS, being admitted to a high-risk ward, and urological manipulation are also significantly associated with an increased risk of infection in KPC-Kp-colonized patients. Our study suggests that in situations of KPC-Kp outbreaks or endemicity appropriate infection control and preventive measures may be implemented during these time windows of increased risk of infection (hand wash, frequent culture screen, isolation, limitation of manipulation, and SBD).

Recent European Society of Clinical Microbiology and Infectious Diseases (ESCMID) consensus guidelines do not recommend routine selective bowel decolonization (SBD) in KPC-Kp-colonized patients (19). It is common for patients undergoing SBD to become recolonized when treatment is discontinued. In addition, SBD increases the risk of producing resistance to the antibiotic used (20, 21). However, knowledge about the periods of high risk of infection identified in this study would allow us to study the efficacy of SBD limited to higher risk time windows. Our group has reported that the use of SBD for 1 month in hospitalized patients undergoing risky procedures (chemotherapy-associated with severe neutropenia, major abdominal interventions, and critically ill patients) reduces mortality (22). However, it would be necessary to design new SBD studies limited to these risk windows in patients with high GRS admitted to centers with outbreaks or high endemicity.

Our study has some important limitations. When patients were colonized in the first rectal swab performed after admission, we could not ascertain how long the patient had been colonized. Furthermore, rectal swab controls may not have been indicated for non-severely ill patients who were discharged and not included in the study. We do not know how many of these patients may have been colonized, so there is an inclusion bias since colonization was only identified in more severely ill patients who were admitted for longer periods. However, the fact that none of the uncolonized, no-screened, or excluded patients developed KPC-Kp infection, may reduce concerns that there was a large pool of colonized, but undetected, patients in this cohort. To carry out a prospective study like this and taking into account that being colonized was a necessary condition for developing an infection, it was necessary to follow up a large number of patients (1310) to finally be able to analyze a much smaller number of colonized patients (166). The higher the incidence and prevalence rates observed in this study compared to other studies (23) may be explained by the fact that our study was carried out in the setting of an outbreak and subsequent endemicity, and only hospitalized patients admitted to higher risk services or undergoing invasive procedures were followed up. This study has allowed us to identify a time window from the timing of colonization that entails a greater risk of developing an infection. This evidence may be incorporated in future studies of prophylaxis or infection control.

## MATERIALS AND METHODS

### Study design and patients.

We conducted an observational, prospective, longitudinal cohort study of hospitalized patients ≥18 years of age undergoing active rectal cultures screening to rule out KPC-Kp colonization at Reina Sofia University Hospital (HURS), a 1000-bed tertiary center in Cordoba, Spain. Patients were included in the cohort (Angel cohort) from July 2012 to November 2017, during an outbreak of KPC-Kp infections that became endemic. KPC-Kp colonization screening was performed by rectal swab culture (the collected specimen was a transport swab) in patients admitted to the intensive care unit (ICU) and hematology unit, patients undergoing abdominal interventions or transplants, patients previously admitted to units affected by the outbreak, and patients sharing a room with a new colonized patient. Patients admitted to the ICU or hematology unit were first screened at admission and weekly during hospitalization. The rest of the patients were screened at admission, when a colonized roommate was detected, or before surgery (when they were transferred to the operating room). Follow-up screening was performed every 2 weeks. For the analysis, day 0 was defined as the date of inclusion in the cohort, when the first rectal swab screening was performed. Patients colonized at day 0 or those who became colonized during hospitalization were included in the cohort. Patients not colonized at day 0 and without at least one positive rectal swab culture during hospitalization were excluded. Patients with an active infection at the time of the first rectal swab (day 0) and those who received intraluminal intestinal decolonization with gentamicin or colistin were also excluded. All consecutive patients who fulfilled inclusion criteria were included in the cohort.

All patients were followed according to the center’s clinical protocols. When the patients were discharged before the end of follow-up, they or their family members were contacted by telephone to ascertain their clinical status. The study was approved by the Ethics Committee of the Reina Sofia University Hospital-IMIBIC (code MOR-ANG-2018-09). All the data obtained were anonymized.

### Variables.

The primary outcome variable was KPC-Kp infection until day 90 of follow-up. Data were collected using a standardized form. Explanatory variables included: (i) demographics: age and sex; (ii) hospitalization-related variables: admission in the previous 6 months, ICU stay during follow-up, admission ward and date of admission; (iii) invasive procedures performed during follow-up: urological manipulation, use of central venous catheter and need for mechanical ventilation during follow-up; (iv) comorbidities: diabetes mellitus, heart failure, chronic obstructive pulmonary disease, kidney disease, neoplasia, neutropenia (<500/μL), Charlson and McCabe indexes; (v) concomitant treatment in the previous month: steroids, antibiotic drugs against Gram-negative bacilli for more than 48 h, and specifically use of carbapenems; (vi) variables related to KPC-Kp colonization: GRS (predictor of bacteremia in KPC-Kp-colonized patients) (10); (vii) variables related to the KPC-Kp infection episode: all-site KPC-Kp infection, days from colonization to infection, INCREMENT-CPE score to evaluate the risk of death (11); and (viii) variables related to mortality: crude mortality, attributable and not attributable to KPC-Kp infection. In addition, the variable “time to colonization” was included (see definition below).

### Definitions.

Rectal colonization by KPC-Kp was defined as isolation of KPC-Kp from a rectal swab in the absence of clinical signs and symptoms of infection. The first day of follow-up was defined as the day that the first screening rectal swab was collected (day 0). The variable time to colonization was considered zero in patients who were colonized at day 0 (patients colonized at start of follow-up). In patients colonized during follow-up, the time from the first negative swab (day 0) to the first rectal swab positive for KPC-Kp was considered.

All Kp-KPC infections were microbiologically tested and defined following the Centers for Disease Control and Prevention criteria (24). The date of infection was defined as the day that the index culture was taken.

### Microbiological studies.

KPC-Kp strains were isolated and characterized following the usual protocols of the center. Plates with selective medium for carbapenemase-producing strains were employed for rectal swab culture screening as follows: ColorexTM KPC plates (RPD Microbiology, Barcelona, Spain) were used from July 2012 to November 2016; CARBA plates (bioMérieux S.A., Marcy-l’Étoile, France) were used from December 2016 to March 2017 and CHROMID CARBA SMART biplates (bioMérieux S.A., Marcy-l'Étoile, France) were used from April 2017 until the end of the study. Identification and antibiogram of isolates were performed from July 2012 to March 2017 using commercial Gram-negative REV.2 WIDER microdilution panels and NC54 combo panels (Siemens Healthcare Diagnostics, Camberley, UK) for the semiautomated WIDER system (Francisco Soria Melguizo S.A., Madrid, Spain) following the manufacturer’s recommendations. Subsequently, MicroScan Gram-negative Combo Panels Type NC53 (Beckman Coulter, CA, USA) was used from March 2017 until the end of the study. The MIC and clinical category for each antibiotic were interpreted from July 2012 to February 2017 using the CLSI cutoff points (25) and from March 2017 until the end of the study using the EUCAST breakpoints for *Enterobacterales* (clinical breakpoint for bacteria v. 6.0 of 2017 and subsequent versions). In some cases, identification was confirmed using a MALDI-TOF (Bruker Daltonik, Bremen, Germany) following the manufacturer’s instructions. Analysis of the sensitivity results established the suspicion of carbapenemase production. KPC production was confirmed by a commercial Xpert Carba-R PCR assay (Cepheid, Sunnyvale, CA, USA) and the KPC K-SeT immunochromatographic assay (Coris BioConcept, Gembloux, Belgium) from July 2012 to March 2017, and the NG-Test Carba 5 immunochromatographic assay (NG Biotech, Guipry, France) from March 2017.

### Statistical analysis.

The results of the analysis were expressed as medians (interquartile range) for the quantitative variables and as percentages for the qualitative variables. Continuous variables were analyzed using the Mann–Whitney *U* test. Categorical variables were compared using the Chi-square test or Fisher’s exact test when indicated. To control the influence of the different hospital wards and the risk of KPC-Kp, wards were classified by TreeNet as being at high or low risk for acquiring KPC-Kp infection (26), considering all other variables; therefore, wards classified as high-risk were those with high infection rate after consideration of patients’ features. A classification and regression trees (CART) analysis (27) was performed to obtain a cutoff point for the variable time to colonization, after which the risk of infection was higher. Differences in the frequency of KPC-Kp infection in the different periods were also analyzed with CART. Logistic regression was performed to study the variables associated with KPC-Kp infection. Cox regression was excluded because the proportional hazards principle was not met. KPC-Kp infection-free survival curves were plotted using the Kaplan–Meier method and compared using the log-rank test. The variable time-to-event in these curves was considered in two different ways: from the date of the first rectal swab until the primary outcome (KPC-Kp infection) occurred and from the date of the first positive KPC-Kp rectal swab to KPC-Kp infection. Censoring was considered both for loss to follow-up of patients, which only occurred in patients who died before developing KPC-Kp infection, or for patients reaching the end of the follow-up period (90 days). R software (version 3.0.1), SPSS 26.0 (SPSS Inc.), and the Salford Predictive Modeler software suite 8.2, which includes CART and TreeNet, were used for the statistical analysis.
