# Supplementary material for: Association between Timing of Colonization and Risk of Developing Klebsiella pneumoniae Carbapenemase-Producing K. pneumoniae Infection in Hospitalized Patients
Source: Microbiol Spectr. 2022 Mar 24;10(2):e01970-21. doi: 10.1128/spectrum.01970-21 (PMC9045231; doi:10.1128/spectrum.01970-21)
Supplement: SUPPLEMENTAL FILE 1 — Supplemental material. Download SPECTRUM01970-21_Supp_1_seq6.pdf, PDF file, 0.2 MB [file spectrum01970-21_supp_1_seq6.pdf]

**Supplementary Table 1. Variables related with the screening protocol**

| Variable | |
|---|---|
| Distribution of swabs per patient, median (IQR). | 5.00 (3.00-7.00) |
| Distribution of time (days)-from-hospitalization to first swabbing, median (IQR). | 2.00 (1.00-6.00) |
| Distribution of number of negative swabs before a positive swab in the patients who become colonized on follow-up, median (IQR). | 2.00 (1.00-2.00) |

**Supplementary Table 2. Distribution of infection types in the two colonization groups.**

| Type of KPC-Kp infection | Colonized at start of follow-up (n=47) | Colonized during follow-up (n=31) |
|---|---|---|
| Bacteraemia | 18 (38,3) | 13 (42,0) |
| Pneumonia | 11 (23,4) | 8 (25,8) |
| Urinary tract infection | 10 (21,2) | 2 (6,4) |
| Abdominal | 3 (6,4) | 5 (16,1) |
| Others | 5 (10,6) | 3 (9,7) |

Data presented as number of patients (percentage). Chi-squared test p=0.34

**Supplementary Figure S1. CART analysis dichotomizing the variable time to colonization and risk of KPC-Kp infection.**

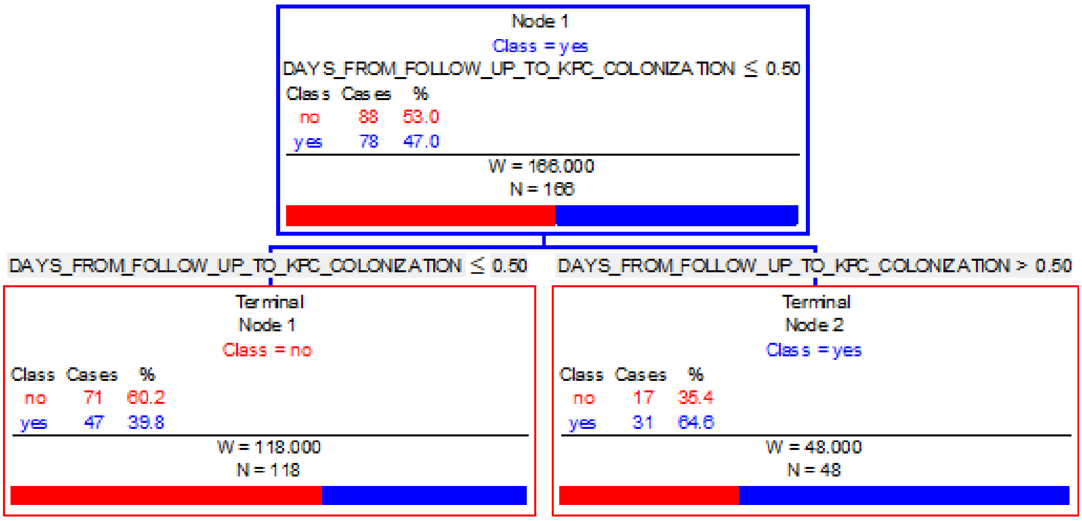


The CART analysis categorizes the continuous variable time to colonization (in days) into a two-category variable: patients who were colonized at day 0 (colonized at start of follow-up) and patients colonized during follow-up (time from the first negative swab to the first rectal swab positive for KPC-Kp > 0 days).

**No = No KPC-Kp infection**

**Yes = KPC-Kp infection**

**Supplementary Figure S2. KPC-Kp infection risk classification by hospital ward using TreeNet (high vs. low risk).**

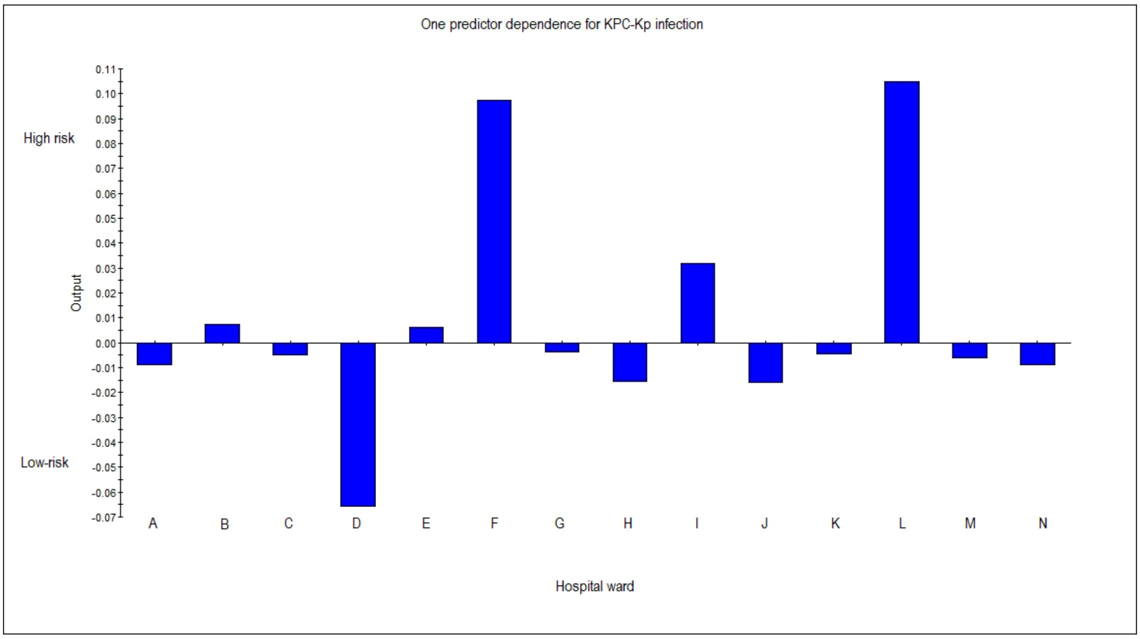

**Supplementary Figure S3. CART analysis to categorize risk of KPC-Kp infection in different patient follow-up periods.**

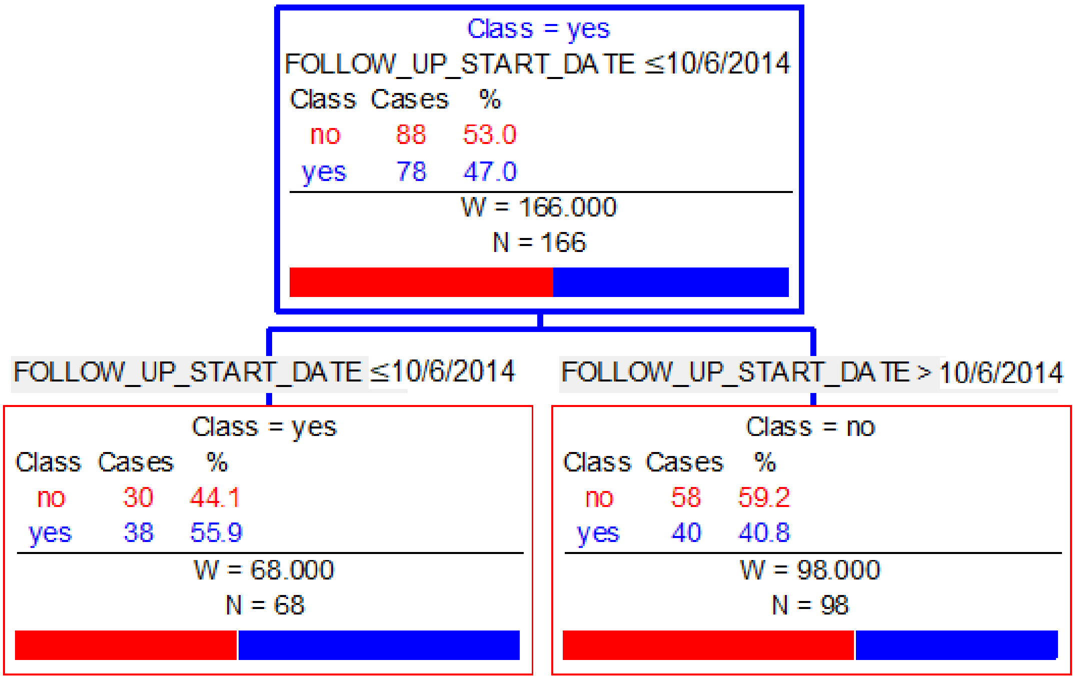


**No = No KPC-Kp infection**

**Yes = KPC-Kp infection**
